# Plummer-Vinson Syndrome in a Pakistani Woman: A Case Report

**DOI:** 10.7759/cureus.83530

**Published:** 2025-05-05

**Authors:** John Pueringer, Evan Garrad, James Love, Anna M Lipowska

**Affiliations:** 1 Internal Medicine, University of Illinois at Chicago, Chicago, USA; 2 Gastroenterology and Hepatology, University of Illinois at Chicago, Chicago, USA

**Keywords:** dysphagia, esophageal web, esophagogastroduodenoscopy (egd), esophagus, iron deficiency anemia, plummer-vinson syndrome

## Abstract

Plummer-Vinson Syndrome (PVS) is characterized by dysphagia, iron deficiency anemia, and esophageal web formation. We discuss the case of a 36-year-old woman from Pakistan who presented with dysphagia for approximately 10 years, cheilitis, and glossitis. Barium esophagram was consistent with a proximal esophageal web. Esophagogastroduodenoscopy (EGD) found a cricopharyngeal stenosis requiring multiple dilations. Initial hemoglobin was 5.0 g/dL, mean corpuscular volume 51.6 fL, ferritin level 1 ng/mL, and iron level <10 μg/dL. She was diagnosed with PVS, treated with iron supplementation, and had resolution of symptoms and dysphagia.

## Introduction

Plummer-Vinson Syndrome (PVS), also known as Paterson-Brown-Kelly syndrome, is a rare and enigmatic condition characterized by a triad of dysphagia, iron deficiency anemia, and esophageal web formation. First described over a century ago, this syndrome has continued to challenge clinicians and researchers alike due to its complex clinical presentation and limited prevalence. PVS classically affects middle-aged Caucasian women, but some cases have been reported in women of Southwest Asian descent [1]. Despite its rarity, PVS remains an important clinical entity, as its early recognition and management can substantially improve the quality of life for affected individuals. Iron supplementation is the primary treatment for PVS, and dysphagia typically improves with iron therapy even before the hematologic abnormalities resolve. Those with advanced and long-standing dysphagia typically require mechanical dilation. This case was previously presented as a meeting abstract at the 2024 American College of Gastroenterology (ACG) Annual Scientific Meeting on October 28, 2024.

## Case presentation

A 36-year-old woman from Pakistan with no significant past medical history was referred to a tertiary care center for evaluation of progressive dysphagia over 10 years, accompanied by cheilitis and glossitis, characterized by a beefy red tongue and a burning sensation involving her tongue and inner left cheek. She reported difficulty swallowing solid foods and pills, often experiencing choking episodes. However, she denied weight loss or loss of appetite. Her only home medication was daily omeprazole for acid reflux.

**Figure 1:**
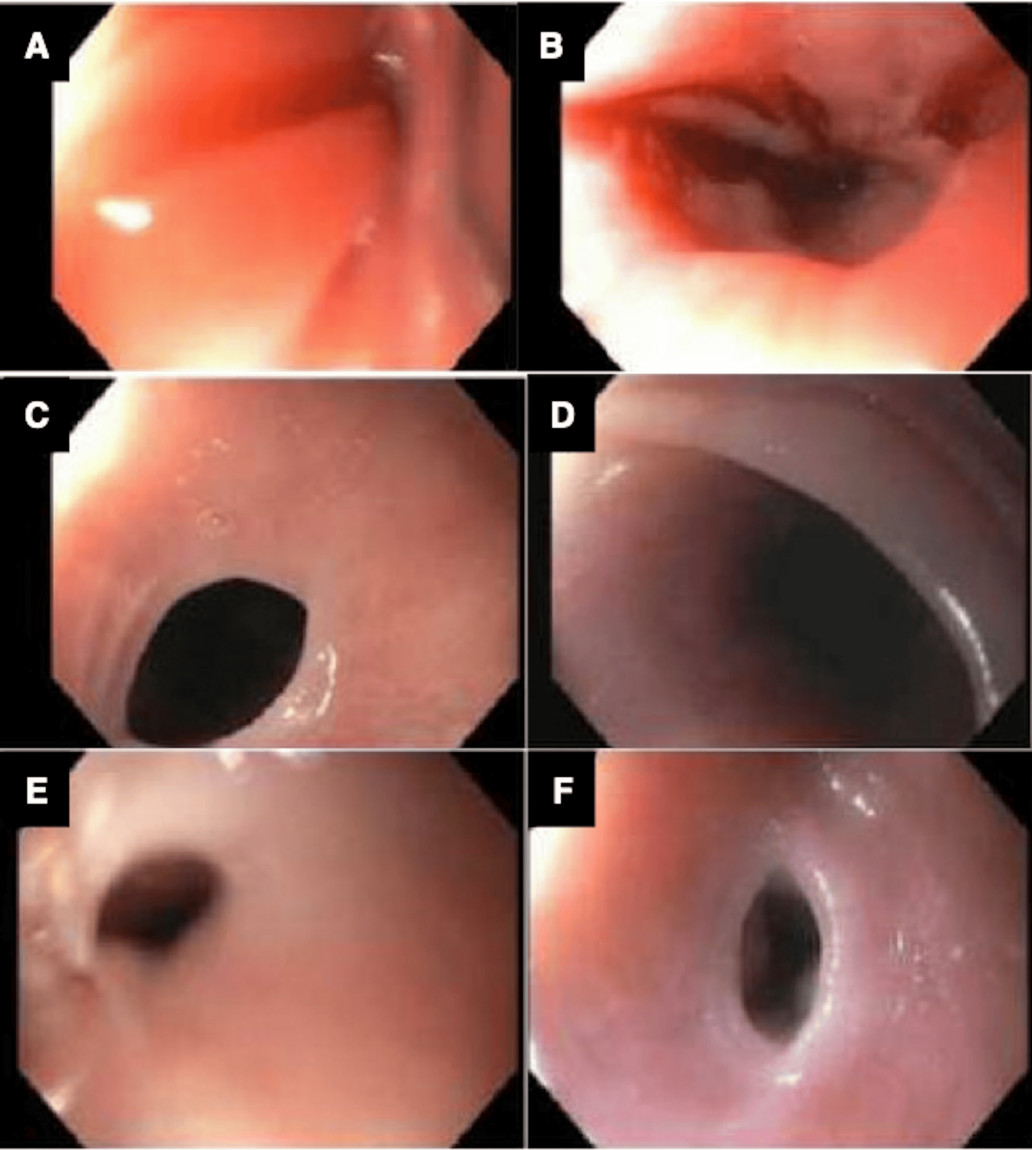
Cricopharyngeal stenosis noted on endoscopy Cricopharyngeal stenosis noted on A) initial endoscopy, and follow-up at B) 2 months; C) 16 months; D) 18 months; E) 24 months; and F) 42 months, all requiring endoscopic Savary dilation, prior to iron therapy. Source: Pueringer et al. [2]. Figure 1 is reproduced with permission from Wolters Kluwer Health, Inc., originally published in American Journal of Gastroenterology, 2024. Copyright © 2024 by The American College of Gastroenterology. Permission has been obtained from Wolters Kluwer Health, Inc. for reproduction in this manuscript.

On physical examination, notable findings included cheilitis and glossitis with a beefy red tongue. A barium esophagram revealed a proximal esophageal web. Esophagogastroduodenoscopy (EGD) demonstrated cricopharyngeal stenosis, necessitating serial endoscopic dilations (Figure 1). High-resolution manometry suggested esophagogastric junction outflow obstruction. EGD biopsies were negative for dysplasia, celiac disease, and eosinophilic esophagitis.

Laboratory evaluation revealed severe microcytic anemia with a hemoglobin level of 5.0 g/dL, mean corpuscular volume (MCV) of 51.6 fL, ferritin of 1 ng/mL, and iron level <10 μg/dL (Table 1). The patient had no history of menorrhagia, hematochezia, or melena. Given the classic triad of dysphagia, iron-deficiency anemia, and esophageal webbing, she was diagnosed with Plummer-Vinson syndrome (PVS) [2].

**Table 1:** Laboratory Values

Laboratory Test	Actual Value	Follow-up Values	Reference Range
Hemoglobin	5.0 g/dL	11.1 g/dL	11.7 - 16.0 g/dL
Mean corpuscular volume	51.6 fL	88.8 fL	80.0 - 99.0 fL
Iron	<10 μg/dL	-	35 - 170 μg/dL
Ferritin	1 ng/mL	-	5 - 116 ng/mL
Total Iron Binding Capacity	514 μg/dL	-	250 - 450 μg/dL

Treatment included blood transfusion, intravenous iron supplementation, and oral iron therapy. One month later, her hemoglobin had stabilized at 7.3 g/dL, and her dysphagia had significantly improved. Further evaluation for etiology of severe iron deficiency anemia was completed, including celiac disease serology, folate, and B vitamin levels, vitamin D levels, and thyroid studies, all of which were normal. Blood smear showed microcytic hypochromic red blood cells with anisopoikilocytosis and hypochromatic microcytes. Follow-up colonoscopy biopsies were benign, with no identifiable source of gastrointestinal blood loss. Additionally, endoscopic evaluation revealed no source of possible iron malabsorption due to celiac disease, atrophic gastritis, and chronic inflammation. The patient was not a candidate for video capsule endoscopy given recurrent esophageal strictures. Repeat EGD biopsies remained negative for dysplasia, celiac disease, and eosinophilic esophagitis.

By the fourth month of follow-up, her hemoglobin had improved to 11.4 g/dL, and her dysphagia had completely resolved.

## Discussion

Esophageal web formation in PVS is thought to be due to oxidative stress from iron-dependent enzyme dysfunction [3]. Repeated injury to epithelia due to iron deficiency leads to atrophy of mucosa and degradation of pharyngeal muscles [4]. The dysfunctional enzymes produce myasthenic changes in the swallowing muscles, leading to web formation [5]. The epithelial layer of the upper alimentary tract is especially susceptible to iron deficiency because of its high cell turnover. Esophageal webs are generally located below the cricopharyngeal muscle and asymmetrically attach to the anterior esophageal wall. Even though iron deficiency may explain dysphagia and webs, PVS is not an inevitable consequence of iron deficiency. Other etiologic factors, including genetic, environmental, and immunological, have been proposed [3]. 

Clinical manifestations of PVS classically include iron deficiency anemia, glossitis, and dysphagia, all of which presented in our patient [6]. Dysphagia commonly occurs in the setting of esophageal webs but can also be seen with proximal and distal esophageal strictures. Glossitis can present as a burning sensation, tongue pain, redness, or swelling, loss of papillae of the tongue, or any new visible tongue lesions. Other physical findings may include splenomegaly, edentia, and brittle nails. The disorder most commonly presents in middle-aged Caucasian women, but has presented in other demographics such as African American women [7], although few cases have been reported in women of Southwest Asian descent.

Medical management of Plummer-Vinson syndrome includes iron supplementation. Dysphagia in many patients resolves with successful iron supplementation [3]. However, dysphagia caused by more advanced disease may require endoscopic dilation in addition to medical management. Commonly used techniques include endoscopic balloon dilatation or Savary-Gilliard dilators [8, 9]. Our patient required multiple endoscopic dilations for adequate treatment. Identifying the underlying cause of iron deficiency is an important part of the evaluation. Our patient underwent an extensive workup for iron deficiency anemia, which was unrevealing for other gastrointestinal pathology or hematologic source. She has no abnormal premenopause menstrual bleeding. Celiac disease serology, folate, B vitamin levels, vitamin D levels, and thyroid studies were normal. Endoscopic evidence for gastrointestinal blood loss and possible iron malabsorption due to celiac disease, atrophic gastritis, and chronic inflammation were all unrevealing. Her Iron deficiency was ultimately thought to potentially arise from nutritional and dietary deficiencies. Since adequate supplementation, her anemia, microcytosis, and dysphagia have resolved. 

## Conclusions

This case underscores the diverse populations affected by Plummer-Vinson syndrome (PVS) and highlights the importance of timely diagnosis and management of this rare condition. It demonstrates successful treatment with iron supplementation, emphasizing the need to investigate potential dietary causes when other pathologies are absent. In refractory cases, endoscopic dilation may be necessary to manage esophageal stenosis.
